# Milligan-Morgan Hemorrhoidectomy vs Stapled Hemorrhoidopexy

**DOI:** 10.5812/kowsar.22517464.3363

**Published:** 2012-01-15

**Authors:** Seyed Mohsen Towliat Kashani, Shaban Mehrvarz, Seyed Morteza Mousavi Naeini, Reza Erfanian

**Affiliations:** 1 Baqiyatallah Research Center for Gastroentrology and Liver disease, Tehran, IR Iran; 2 Trauma Research Center, Baqiyatallah University of Medical Sciences, Tehran, IR Iran

**Keywords:** Surgical Procedures, Milligan Morgan, Stapled Hemorrhoidopexy

## Abstract

**Background:**

The stapled hemorrhoidopexy (SH) is a procedure for prolapse and hemorrhoids . At first SH seemed to be a good alternative for the Milligan Morgan (MM) hemorrhoidectomy and preliminary results in early 2000 confirmed it. However, further studies and evaluation of long-term results showed poorer outcomes.

**Objectives:**

This study aimed to evaluate and compare the results of these 2 surgical procedures in terms of recovery, improvement of symptoms and incidence of complications.

**Materials and Methods:**

This study was conducted from April 2008 to August 2010. A total of 80 patients were divided into 2 groups of 40 each. In the SH group, there were 24 males (60%) and 16 females (40%) with a mean age of 48 ± 12.5 yrs. In the MM group, there were 30 males (75%) and 10 females (25%) with a mean age of 50.6 ± 17.3 yrs. Patients with grade 3 and 4 prolapsed hemorrhoids were entered in the study. Data were entered using SPSS software and analyzed using t-test and Chi-square test.

**Results:**

The two groups had no significant difference in terms of age or sex. Duration of surgery was 35 ± 7 minutes in the SH and 23.6 ± 13.5 minutes in the MM group. This difference was statistically significant (P = 0.000). Post-operative pain and complete pain relief was slightly lower in the MM group (not significant). Hospital stay was significantly longer in the MM group (P = 0.003). Return to work was similar in both groups. Three patients in the SH group (7.5%) and 2 in the MM group (5%) had hemorrhoid recurrence.

**Conclusions:**

Both techniques are efficient treatment methods for grade III and IV hemorrhoids and are associated with greater than 95% recovery rate. Overall, outcomes are the same in both techniques. Lower postoperative pain was the only advantage of SH over MM technique.

## 1. Background

Stapled hemorrhoidopexy (procedure for prolapse and hemorrhoid, PPH) was first introduced by Longo in 1998. At first it seemed to be a good alternative for the Milligan Morgan hemorrhoidectomy and preliminary results in early 2000 confirmed this (1, 2). However further studies and evaluation of longterm results showed a poor outcome (3, 4). The most important shortterm results expected from hemorrhoid surgery include recovery and elimination of symptoms like bleeding, prolapse and pain. Longterm complications due to hemorrhoidectomy include recurrence of mucosal prolapse, incontinence and problems due to anal stenosis. At our center, hemorrhoid surgery used to be performed via conventional procedures. More recently, stapler has been used for treatment of prolapsed hemorrhoids. Stapled hemorrhoidopexy (SH) seemed to be a new, less invasive method and several studies reported favorable outcomes (5, 6).

## 2. Objectives 

This study aimed to evaluate and compare the results of these 2 surgical procedures in terms of recovery, improvement of symptoms and incidence of complications.

## 3. Materials and Methods

This study was conducted from April 2008 to August 2010. A total of 80 patients were divided into 2 groups of 40 each. In the SH group, there were 24 males (60%) and 16 females (40%) with a mean age of 48 ± 12.5 yrs. In the MM group, there were 30 males (75%) and 10 females (25%) with a mean age of 50.6±17.3 yrs. Patients with grade 3 and 4 prolapsed hemorrhoids who were candidates for surgery in the colorectal clinic of our hospital entered the study. The exclusion criteria were as follows:

1. Positive history of anorectal surgery
2. Having rectal prolapse
3. Patients with fissure or fistula

The study protocol was approved by the ethics committee of our university.

First, type and method of surgical procedure (SH) was thoroughly explained to the patients and then if they were willing to participate in the study they were placed in the SH group . If they were not interested to undergo this type of procedure, they were placed in the conventional surgery group (MM). Afterwards, patients entered the research project and their data were recorded in specific questionnaires. All surgical procedures in both techniques were performed by experienced surgeons under spinal anesthesia with the patient in lithotomy position. For patients whom were selected for stapled hemorrhoidopexy (SH), 1 gram ceftriaxone injection and 50 mg intravenous metronidazole were administered preoperatively. No antibiotic was administered for patients in the MM group.

Patients were visited and examined on the first postoperative day and one week after surgery in the colorectal surgery clinic. Follow ups were performed 1, 6 and 12 months later via phone. Patients were also visited by the surgeon at the clinic. Post-operative pain was measured according to Visual Analog Scale. For pain scale 1-3 oral acetaminophen, for scale 4-5 acetaminophen codeine and for scale 6-10 intravenous pethidine was administered. Psyllium powder and magnesium hydroxide syrup 8% were administered daily postoperatively. Data were entered using SPSS software and analyzed using t-test and Chi-square test.

## 4. Results 

A total of 80 patients were divided into 2 groups of 40 each according to patient preference. In the SH group, there were 24 males (60%) and 16 females (40%) with a mean age of 48 ± 12.5 yrs. In the MM group, there were 30 males (75%) and 10 females (25%) with a mean age of 50.6 ± 17.3 yrs. The two groups had no significant difference in terms of age or sex. Ninety percent of patients had grade III and 10% of patients had grade IV hemorrhoids. The most important indications for surgery were prolapse in 18 patients (22.5%) and bleeding and prolapse in 62 cases (77.5%). Table 1 shows the grading of hemorrhoids in patients. Duration of surgery was 35 ± 7 minutes in the SH and 23.6±13.5 minutes in the MM group. This difference was statistically significant (P = 0.000).

**Table 1.** Hemorrhoid Grading and Number of Patients in Each Group

Grade	MM no, %	SH no, %
III	33, 82.5	36, 92.5
IV	7, 17.5	4, 7.5
TOTAL	40, 100	40, 100

Severity of post-operative pain according to VAS was 4 ± 1.7 in the SH and 4.6 ± 2.3 in the MM group. Although the severity of pain (pain scale) was lower in the SH group, this difference was not statistically significant (P = 0.166).Duration of hospital stay after the surgery was 1.7 ± 0.6 days in the SH and 1.27 ± 0.6 days in the MM group. This difference was statistically significant (P = 0.003). Time required to achieve complete pain relief was 11.6 ± 10 days in the SH and 13 ± 12.2 days in the MM group. This difference was not statistically significant (P = 0.583).Time to return to work was almost similar in both groups (6.95 ± 3.6 days vs 6.13 ± 4.2 days). The mean follow up period in both groups was 13 ± 5.7 months. At the end of the follow up period, 3 patients in the SH group (7.5%) and 2 in the MM group (5%) had hemorrhoid recurrence. The difference in this regard was not statistically significant between the 2 groups. Full recovery was achieved in 92.5% of the cases in SH and 95% of the cases in the MM group. This difference was not significant either. Other minor complications observed post operatively included transient incontinence, 1 case of perianal abscess, and 1 case of urinary retention. Anal stenosis, or permanent incontinence, did not occur in any of the cases.

## 5. Discussion

In the MM technique hemorrhoidal tissue is completely excised but in SH hemorrhoid is not actually removed and in fact hemorrhoidopexy is performed. This explains the higher rate of recurrence in the SH group (7.5% vs 5%). Considering the obtained results, it seems that stapled hemorrhoidopexy has no advantage over MM in terms of final outcome. Duration of SH surgery is longer but patients are somehow more comfortable after surgery. Our study results in terms of severity of postoperative pain and time to return to work were in agreement with those of Mattana (7). In terms of duration of surgery, duration of hospitalization and return to work and normal daily activities our study results were different from those of Nisar (8) and Tjandra (9).

In terms of general recovery our SH surgery results were in accord with those of Gravie (10) and Tjandra (9). MM technique however in our study had a 95% success rate which was lower than the rate mentioned in above studies. Both SH and MM surgical techniques are efficient treatment methods for grade III and IV hemorrhoids and are associated with good outcome and more than 95% recovery rate. Overall, general outcome has reported to be the same in both techniques in many studies including ours. The only advantage of SH over MM technique is that it is associated with relatively less postoperative pain. However, extensive studies with longterm follow ups showed that SH has a longterm recurrence rate higher than that of MM hemorrhoidectomy (11). Considering the extra costs related to purchasing the stapler and longer hospitalization, it looks like MM is still the method of choice in most cases.
